# Structural Basis of the Chromodomain of Cbx3 Bound to Methylated Peptides from Histone H1 and G9a

**DOI:** 10.1371/journal.pone.0035376

**Published:** 2012-04-13

**Authors:** Jianbin Ruan, Hui Ouyang, Maria F. Amaya, Mani Ravichandran, Peter Loppnau, Jinrong Min, Jianye Zang

**Affiliations:** 1 Hefei National Laboratory for Physical Sciences, Microscale and School of Life Sciences, University of Science and Technology of China, Hefei, Anhui, People's Republic of China; 2 Key Laboratory of Structural Biology, Chinese Academy of Sciences, Hefei, Anhui, People's Republic of China; 3 Structural Genomics Consortium, University of Toronto, Toronto, Ontario, Canada; 4 Department of Physiology, University of Toronto, Toronto, Ontario, Canada; Université Paris-Diderot, France

## Abstract

**Background:**

HP1 proteins are highly conserved heterochromatin proteins, which have been identified to be structural adapters assembling a variety of macromolecular complexes involved in regulation of gene expression, chromatin remodeling and heterochromatin formation. Much evidence shows that HP1 proteins interact with numerous proteins including methylated histones, histone methyltransferases and so on. Cbx3 is one of the paralogues of HP1 proteins, which has been reported to specifically recognize trimethylated histone H3K9 mark, and a consensus binding motif has been defined for the Cbx3 chromodomain.

**Methodology/Principal Findings:**

Here, we found that the Cbx3 chromodomain can bind to H1K26me2 and G9aK185me3 with comparable binding affinities compared to H3K9me3. We also determined the crystal structures of the human Cbx3 chromodomain in complex with dimethylated histone H1K26 and trimethylated G9aK185 peptides, respectively. The complex structures unveil that the Cbx3 chromodomain specifically bind methylated histone H1K26 and G9aK185 through a conserved mechanism.

**Conclusions/Significance:**

The Cbx3 chromodomain binds with comparable affinities to all of the methylated H3K9, H1K26 and G9aK185 peptides. It is suggested that Cbx3 may regulate gene expression via recognizing both histones and non-histone proteins.

## Introduction

The family of Heterochromatin protein 1 (HP1) proteins is a family of highly conserved heterochromatin-associated non-histone chromosomal proteins [1], which has important functions in nucleus. These functions include gene activation or repression [1], [2], [3], [4], regulation of binding of cohesion complexes to centromere [5], [6], [7], sequestration of genes to nuclear periphery [8], and heterochromatin formation and propagation [9], [10]. Orthologs of HP1 proteins have been indentified in yeast, nematode, insects, chicken, mammals and plants [11]. In mammals, there are three paralogs of HP1, HP1α, HP1β and HP1γ (also named as Cbx5, 1 and 3, respectively) [11]. Members of the HP1 family are characterized by a typical domain architecture, comprising of an N-terminal chromodomain (CHD) and a C-terminal dimerization chromo shadow domain (CSD) separated by a poorly conserved hinge region (H) [11], which is different from the Polycomb subfamily of proteins (Cbx2, 4, 6, 7, and 8 in human) [12], [13]. HP1 proteins function as structural adapters which assemble a variety of macromolecular complexes via either N-terminal chromodomain or C-terminal chromo shadow domain [14].

To date, chromodomain has been found in a number of other non-HP1 proteins [1]. In mammals, chromodomain-containing proteins are responsible for gene regulation related to chromatin remodeling and formation of heterochromatin [15]. It has been reported that HP1 proteins mediate gene silencing by dynamic association of the chromodomain with methylated histone tails [16], [17]. Recently, several structures of chromodomains of human HP1 homologues in complex with methylated histone peptides have been solved. Structural analysis of these Cbx proteins indicates that human Cbx1, -3, and -5 preferentially recognize H3K9me3 in a similar manner, which has been observed in *Drosophila* HP1 [12]. In addition to methylated histone H3, HP1 proteins have also been found to interact with numerous other proteins. Some of these HP1 interacting partners are histone methyltransferase Clr4/Suv39 [9], inner nuclear membrane proteins [18], DNA methyltransferase DIM-2 [19], methyl CpG binding proteins [20], and the origin recognition complex protein ORC2 [21]. In addition, recent studies have shown that HP1 proteins can directly bind to not only methylated Lys26 of histone H1 [22] but also automethylated Lys185 of histone methyltransferase G9a via its chromodomain [23]. Lys26 in histone H1.4 is di-methylated next to a phosphorylation site at Ser27 [24]. Though it has been reported that HP1 proteins are involved in methyl-H1K26- and G9a-HP1-mediated gene silencing [25], [26], the molecular mechanisms are still unknown.

In this study, we determined the two crystal structures of the human Cbx3 (HP1γ) chromodomain in complex with methylated histone H1 peptide and histone methyltransferase G9a peptide to elucidate the structure basis for the recognition of Cbx3 to those two proteins. Our structural analysis and biochemical data unveiled that Cbx3 chromodomain binds to H1K26me2 peptide and G9aK185me3 peptide via a conserved recognition mechanism with comparable binding affinities compared to the reported structure of Cbx3-H3K9me3 complex [12]. However, significant differences were still observed, especially for the conformation of H1K26me2 peptide. Thus, our results provided structural evidences that Cbx3 may regulate gene expression via recognizing both histones and non-histone proteins.

## Results

### Cbx3 chromodomain recognizes methylated peptides from histone H1 and G9a

**Figure 1:**
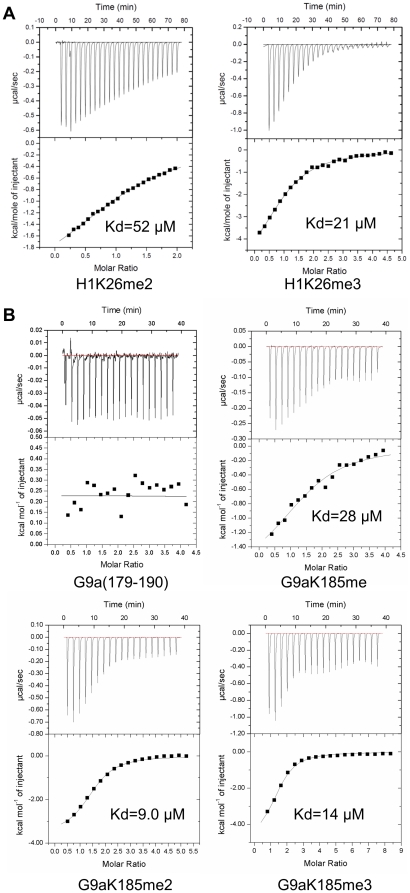
Human Cbx3 chromodomain binds to methylated histone H1K26 and G9aK185. ITC data for Cbx3 chromodomain binding to (A) H1K26 peptides (residues 18–29) and (B) G9aK185 peptides (residues 179–190). Lower panel show fit to a one-site binding model to the binding isotherms.

Chromodomain has been identified to be a methyl-lysine binding motif involved in transcription regulation [27], [28]. Many chromodomain-containing proteins, such as HP1 proteins and Polycomb group proteins, were reported to recognize methylated histone tails [12], [13], [17], [29]. Besides methylated histone H3K9, Cbx3, also known as HP1γ, can also bind to methylated Lys26 of histone H1 and methylated Lys185 of G9a [12], [22], [23]. To elucidate the binding affinity of Cbx3 chromodomain to these two methylated sites, isothermal titration calorimetry (ITC) assay was performed using histone H1K26 and G9aK185 peptides bearing different lysine methylation states as substrates. As expected, we found that Cbx3 chromodomain did not exhibit detectable binding to either the H1K26 or G9aK185 peptide without modification (Table 1 and Fig. 1). However, Cbx3 chromodomain was found to possess strong binding affinity to both di- and tri-methylated H1K26, with a stronger binding affinity for the tri-methylated mark (*K_d_* values of 21 µM for H1K26me3 compared to 52 µM H1K26me2) (Table 1 and Fig. 1A). On the other hand, Cbx3 chromodomain can bind to G9aK185 peptides with comparable binding affinity regardless of their methylation states (Table 1 and Fig. 1B). Furthermore, we found that Cbx3 chromodomain binds to both methylated H1K26 and G9a peptides with comparable dissociation constant values compared to tri-methylated H3K9 peptides (*K_d_* = 15 µM) [12] (Table 1).

**Table 1:** Peptide binding specificity of human Cbx3 chromodomain.

*K_d_*_ peptide_ (µM)					
	Peptide sequence	unmodified	me1	me2	me3
H1.4(18–29)	TPVKKKARK^26^SAG	N/B*	N/B	52	21
G9a(179–190)	KVHRARK^185^TMSKP	N/B	28	9.0	14

* N/B: No binding was detected.

Overall, our binding assay demonstrated that Cbx3 chromodomain binds with comparable affinity to all of the methylated H3K9, H1K26 and G9aK185 peptides. Kaustov, L. *et al* have proposed that chromodomain from Cbx proteins appear capable of binding to an alternative “ARKS/T” motif [12], which is shared in all the H3K9, H1K26 and G9aK185 peptides. It seems that Cbx3 chromodomain binding to these methylated sites is driven by the “ARKS/T” motif, and molecular mechanism of the interaction should be conserved.

### Structure basis for Cbx3 binding to methyl-histone H1K26 and -G9a-K185

To unveil the molecular mechanism of the Cbx3 chromodomain interacting with methylated histone H1K26 and G9aK185, Cbx3 chromodomain (residues 29–86, referred to as Cbx3) in complex with histone H1K26me2 (residues 18–29) peptide and G9a-K185me3 (residues 179–190) peptide were crystallized, respectively. The crystals were diffracted to 1.8 Å and 2.4 Å resolution, respectively. The structures were solved by molecular replacement method using the structure of *Drosophila* Polycomb chromodomain (PDB code: 1PDQ) as template. The quality of the X-ray diffraction data and the structure refinement parameters are shown in Table 2.

**Table 2:** X-ray Data collection and refinement statistics.

PDB Code	3TZD	3DM1
**Data collection**		
Crystals	Cbx3-H1K26me2	Cbx3-G9aK185me3
Space group	*I*23	*P*3_2_21
Cell dimensions		
*a*, *b*, *c* (Å)	92.2, 92.2, 92.2	83.7, 83.7, 110.3
α, β, γ (°)	90, 90, 90	90, 90, 120
Wavelength (Å)	1.5418	1.2827
Resolution (Å)	40.00-1.81(1.86-1.80)^a^	40.00 – 2.40(2.49 – 2.40)
*R*_merge_ (%)^b^	8.6(51.2)	8.8(83.6)
*I*/σ*I*	13.9(7.6)	15.1(4.4)
Completeness (%)	98.3(82.7)	99.9(100.0)
Redundancy	20.7(19.6)	10.5(10.4)
No. unique reflections	12066	18022
**Refinement**		
Resolution (Å)	40.00-1.81	39.10 – 2.40
No. reflections	11,460	17,856
*R*_work_ c/*R*_free_ d	19.9/23.1	22.0/26.7
No. atoms		
Protein	1,225	2,019
Water	19	91
Average B-factors (Å^2^)		
Protein	18.8	43.3
Water	24.8	44.5
R.m.s. deviations		
Bond lengths (Å)	0.019	0.021
Bond angles (°)	1.629	1.826
Ramachandran plot^e^	95.0	94.2
Most favored regions (%)	5.0	5.8
Additionally allowed regions (%) Outliers (%)	0.0	0.0

a The values in parentheses refer to statistics in the highest shell.

b Rmerge = |Ii−<I>|/|Ii| where Ii is the intensity of the ith measurement, and <I>is the mean intensity for that reflection.

c Rwork = Σh|Fo(h)−Fc(h)|/ΣhFo(h), where Fo and Fc are the observed and calculated structure factor amplitudes, respectively.

d Rfree was calculated with 10% of the reflections in the test set.

e Categories were defined by MolProbity.

**Figure 2:**
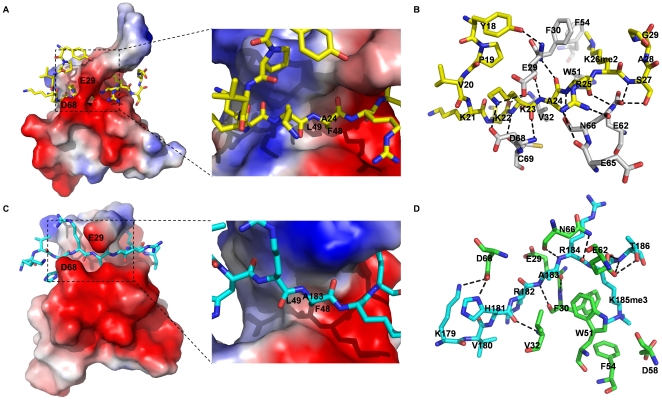
Structure basis for Cbx3 binding to methylated histone H1K26 and G9aK185 peptide. (A and C) Electrostatic surface depiction of human Cbx3-histone H1K26me2, and Cbx3-G9aK185me3 complex. Peptide substrates are shown in a stick mode. Surfaces with positive electrostatic potential are blue, and negative potential are red. The side chain of residue H1A24 (G9aA183) inserts into the small hydrophobic pocket formed by Phe48 and Leu49 of human Cbx3. The size of the pocket is only sufficient to accommodate a methyl group but not other residue side chains. (B and D) Binding of histone H1 peptide and G9a peptide in the binding groove of Cbx3 chromodomain, respectively. Hydrogen-bonds are shown as dashed lines. Yellow: histone H1 peptide; Gray: Cbx3 chromodomain in Cbx3-histone H1K26me2 complex. Cyan: G9a peptide; Green: Cbx3 chromodomain in Cbx3-G9aK185me3 complex.

The overall structures of Cbx3 in complex with histone H1K26me2 peptide and G9a-K185me3 peptide are shown in Fig. 2A and 2C, respectively. Both structures adopt canonical chromodomain fold and bind methyl-lysine containing peptides in a manner similar to *Drosophila* HP1. Six amino acid residues of both peptides, “KKAR(K^26^me2)S” of histone H1 and “HRAR(K^185^me3)T” of G9a (referred to as binding motif), are buried in the binding groove of Cbx3 (Fig. 2A and 2C). In this region of both peptides, the carbonyl groups of H1K22 and G9aH181 form hydrogen bonds with Val32 of Cbx3, respectively. The imidazolyl group of residue G9aH181 forms an additional hydrogen bond with Asp68 of Cbx3 in Cbx3-G9aK185me3 complex. Residue H1K23 forms both main-chain and side-chain hydrogen bonds and salt bridges with Glu29, Asp68 and Cys69 (Fig. 2B). H1A24 is anchored in the groove by Glu29 and Phe30 of Cbx3 through main chain hydrogen-bond interactions (Fig. 2B). Similarly, G9aA183, the counterpart of H1A24 is tethered in the same way (Fig. 2D). Besides, the side chains of both alanine residues are buried in a small hydrophobic pocket formed by Phe48 and Leu49, the size of which is only sufficient to accommodate a methyl group, consistent with the observation from the structure of Cbx3-H3K9me3 (Fig. 2A and 2C) [12]. In addition, H1R25 and H1S27 (corresponded to G9aR184 and G9aT186 in binding motif) form several hydrogen bonds and salt-bridges with Asn66 and Glu62 of Cbx3, respectively (Fig. 2C and 2D).

In the structure of Cbx3-H1K26me2 complex, the dimethyl-ammonium of K26 is accommodated in a hydrophobic pocket formed by three aromatic residues, Phe30, Trp51, and Phe54. The side chain of dimethylated Lys26 is stabilized by cation-π and van der Waals interactions within the aromatic cage (Fig. 2B). In Cbx3-G9aK185me3 complex structure, the side chain of tri-methylated Lys185 is anchored in the same aromatic cage. In addition to cation-π and van der Waals interactions found in the complex structure of Cbx3-H1K26me2, the tri-methylated lysine residue of G9aK185me3 in Cbx3-G9aK185me3 complex forms salt bridge with Asp58 on the other side of the methyl-lysine binding pocket (Fig. 2D). Generally, we conclude that Cbx3 chromodomain specifically bind methylated histone H1K26 and G9aK185 peptides through a conserved mechanism: 1) The side chain of the methyl-lysine residue is positioned in a cage consisting of three aromatic residues, which is similar to other methyl-lysine binding modules [30], [31], [32]; 2) Residue alanine at the −2 position of the methylated peptide is anchored in the binding groove through main-chain interactions with Cbx3. Moreover, the side chain of the alanine is buried in a small hydrophobic pocket, which is a strict requirement for the recognition between Cbx3 and its binding partner; 3). The remaining residues of the peptide interact with the chromodomain binding groove via hydrogen bonds, salt bridges and van der Waals interactions.

### Comparison of three structures of Cbx3 chromodomain binding to methylated histone H3, H1 and G9a peptides

**Figure 3:**
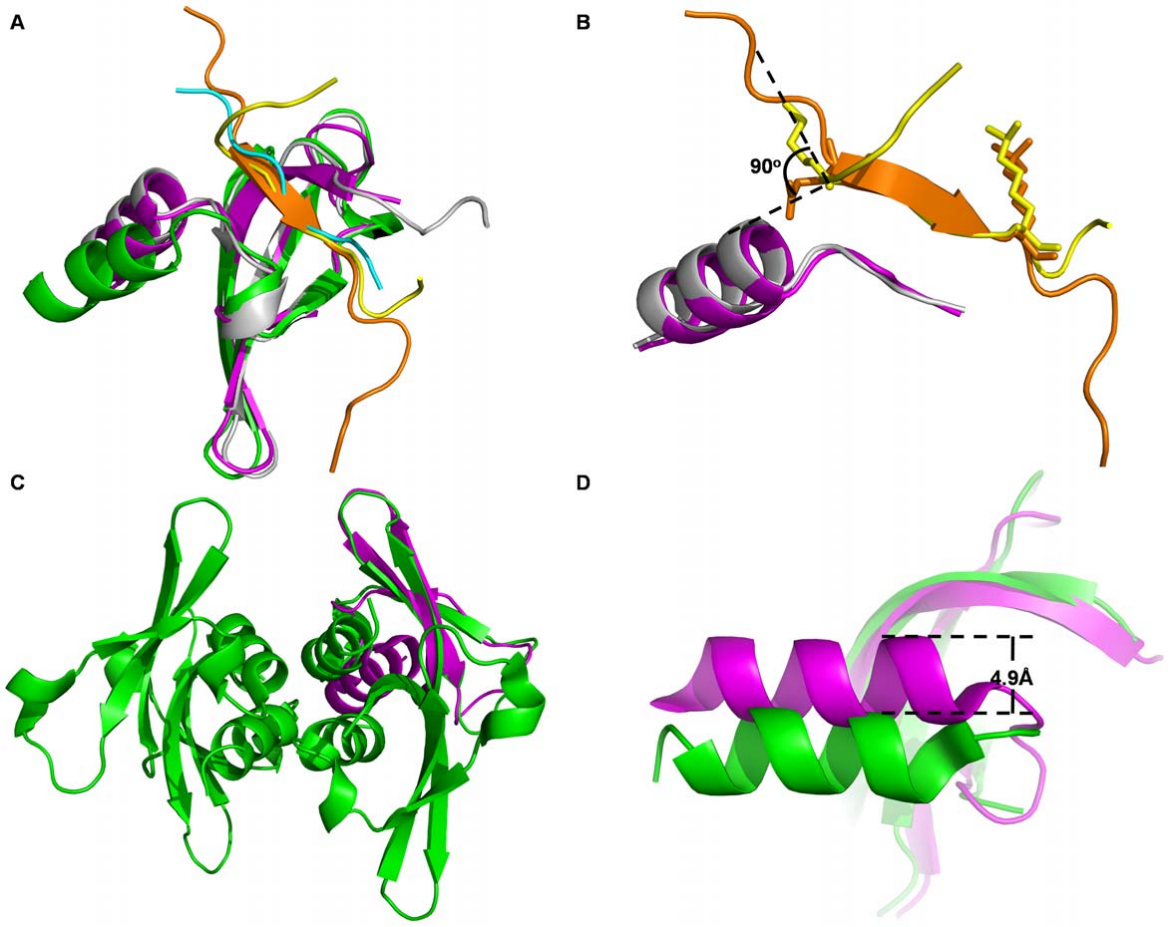
Comparison of three structures of Cbx3 chromodomain binding to methylated histone H3, H1 and G9a peptides. (A) Superposition of human Cbx3 chromodomain in complex with methylated histone H1 peptide (yellow), histone H3 peptide (orange), G9a peptide (cyan), Cbx3 chromodomains are colored as magenta, gray and green, respectively. (B) Superposition of histone H1 peptide (yellow), histone H3 peptide (orange). (C) Structure of Cbx3-H3K9me3 complex (magenta) was superposed to one protomer of the tetramer of Cbx3-G9aK185me3 complex (green) formed in one asymmetric unit. (D) The α helix (residues 70 to 79) of the chromodomain in the structure of Cbx3-G9aK185me3 complex (green) shifts 4.9 Å away from its counterpart in the structures of Cbx3-H3K9me3 (magenta).

We then compare the two structures of Cbx3 in complex with methylated histone H1 and G9a peptides with the structure of Cbx3-H3K9me3 complex (PDB code: 2L11) [12]. Binding of both histone H1 and G9a peptides do not induce significant conformational change of the binding site of the chromodomain compared to the structure in complex with H3K9me3 peptide (Fig. 3A). The root-mean-square deviations (RMSD) are 0.7 Å and 1.8 Å for the aligned C_α_ atoms of the complex structures of Cbx3-H1K26me2 and Cbx3-G9aK185me3 relative to Cbx3-H3K9me3, respectively. The conformations of the binding motifs, “KKAR(K^26^me2)S” of histone H1 and “HRAR(K^185^me3)T” of G9a, are also very similar to their counterpart, “QTAR(K^9^me3)S” of H3, in the complex structure of Cbx3-H3K9me3, with an RMSD of 0.47 Å and 0.02 Å, respectively (Fig. 3A).

In spite of the overall conformational similarity among the structures of Cbx3 in complex with methylated histone H3, H1, and G9a peptides, there are still variations around the interface where peptide substrate interacts with chromodomain. In contrast to H3K9me3 peptide in the structure of Cbx3-H3K9me3 complex, both of the methylated histone H1 and G9a peptides do not form β strand (Fig. 3A). In the structure of Cbx3-H1K26me2 complex, the main chain of Lys22 at position −4 of the binding motif adopts a rotation of about 90° relative to the structures of both the G9a peptide and H3 peptide. So that the side chain of Lys22 can be precisely inserted into the binding groove (Fig. 3A and 3B). As a result, the conformation of amino acid residues before the “KKAR(K^26^me2)S” of the methylated histone H1 peptide shifts from the positions of its counterparts in histone H3 peptide (Fig. 3B). The hydrogen bond formed by the side chain of Tyr18 of histone H1 peptide with Glu29 of Cbx3 stabilizes such conformation (Fig. 2B). When compared with Cbx3-H3K9me3 complex, the α helix (residues 70 to 79) of Cbx3 in the structure of Cbx3-G9aK185me3 complex moves 4.9 Å away from the position of its counterpart in Cbx3-H3K9me3 complex, which may result from crystal packing artifact (Fig. 3C and 3D). Because there are four Cbx3-G9aK185me3 complex in one asymmetric unit and the α helices from the four protomers assemble into a four-helix bundle (Fig. 3C). Thus, we concluded that though Cbx3 chromodomain binds to H1K26me2 peptide and G9aK185me3 peptide similarly to H3K9me3, significant differences were still observed, especially for the conformation of H1K26me2 peptide.

## Discussion

Though HP1 was firstly identified to be transcriptional repressor, more and more studies have uncovered HP1 as a functionally multifaceted protein involved in not only heterochromatin formation and gene silencing, but also transcriptional elongation, centromeric sister chromatid cohesion, telomere maintenance and DNA repair [10], [33], [34]. Numerous biomacromolecules, including proteins and nucleic acids, have been identified to be HP1-binding partners [33]. Tri-methylated lysine 9 residue of histone H3 (H3K9me3) is one of the most intensely studied binding sites of HP1. Recent research indicated that binding of HP1 to H3K9me3 marks provided a platform for a number of interacting partners which would directly/indirectly read out the information coded by H3K9me3 [23], [35], [36].

It is interesting that Cowell, I. G. *et al* found that HP1 was able to tethered to chromatin regions lacking of methylated H3K9 [35]. The observations suggested that the association of HP1 with such chromatin regions does not require H3K9me3. To explain the observations, methylated lysine 26 residue of histone H1 was considered to be required for the binding of HP1 [22]. Histone H1 is a linker histone which binds to the “nucleosome” from outside and facilitates further compaction of chromatin [37]. In humans, there are about 10 variants of histone H1, and many variants of histone H1 follow a cell type or tissue specific expression pattern [37], [38]. Lysine 26 is present in all somatic H1 subtypes as well as testis specific H1 histone H1t, though the N termini of histone H1 are poorly conserved. However, only H1.4 is methylated at lysine 26 and shares the “ARK^26^S/T” motif [25], [39]. Thus we proposed that specific binding of Cbx3 chromodomain to H1K26me2 should play an important role in H1K26me2 dependent gene regulation, and even development and differentiation.

Among HP1-binding partners that function in the stability of the higher-order structure of heterochromatin and gene silencing, many of those are histone methyltransferases (HMTases). HP1 firstly bound to methylated H3K9 to establish a platform which in turn recruits more histone H3K9 methyltransferase. This propagation path of heterochromatin formation is called “self-sustaining loop” [10]. Several histone H3K9 methyltransferases have been reported to directly interact with HP1, including Clr4, SUV39H1 and G9a [9], [23], [40]. However, interaction between G9a and HP1 is different from that between other HMTases and HP1 [9], [23], [40]. Consistent with previous reports [23], our research provided structural evidence that Cbx3 chromodomain interacts with automethylated K185 of G9a in a similar molecule mechanism compared with H3K9me3.

In summary, we determined the structures of Cbx3 chromodomain in complex with two different methylated peptides, H1K26me2 and G9aK185me3 respectively. Our structural data showed that Cbx3 chromodomain bound to both the methylated peptides in a conserved molecule mechanism with comparable binding affinity compared to H3K9me3 peptide.

## Materials and Methods

### Protein expression and purification

The chromodomain of Cbx3 (29–86) was subcloned into a pET-28a-MHL vector. The protocol for protein expression and purification is similar to that described before [41]. The N-terminal histag was cleaved from Cbx3 chromodomain by the addition of 0.05 mg of TEV protease per milligram of Cbx3, followed by dialysis at 4°C for 12 h to remove imidazole. The sample was then passed through a Ni-NTA column and the flow-through was collected and further purified by size exclusion chromatography (Superdex 75, GE Healthcare). Purified Cbx3 chromodomain was collected and concentrated to 26 mg/ml in 50 mM Tris–HCl, pH 8.0 and 100 mM NaCl and 1 mM TCEP.

### Isothermal titration calorimetry

Isothermal titration calorimetry measurements were performed as reported previously [42]. Experiments were carried out by injecting 60 µl of peptide solution (1–2 mM) into a sample cell containing 40–100 mM protein of Cbx3 chromodomain in 20 mM Tris–HCl, pH 7.5, 150 mM NaCl. The ITC measurements were fit to a one-site binding model using Origin Software (MicroCal Inc.)

### Protein crystallization, X-ray diffraction data collection and structure determination

CBX 3 (26 mg/ml) and the trimethylated lysine peptide were mixed in the ratio 1∶1.2 and incubated overnight at 4°C and the hanging drop was set up against the well solution of 40% PEG 550MME at 298 K at 1∶1 ratio. Crystals appeared after 4 days and were flash frozen in liquid nitrogen and the data was collected to 2.4 Å and 1.8 Å, respectively.

The data was processed with HKL2000 and scaled with Scalepack [43]. Results can be found in the files den_out.pdf and scale_log.pdf attached. The data integrated and scaled in the space group *P*322_1_ was converted to mtz using the programs truncate and unique. Molecular replacement was then performed using the program Amore [44] using a model of the structure of *Drosophila* Polycomb chromodomain (PDB code: 1PDQ). A good solution with four molecules in the asymmetric unit was obtained. The program RESOLVE [45] was used to improve the phases obtained with the refined Amore model and ARP_WARP [46] built 95% of the molecule.

CBX3 structure was obtained by molecular replacement and traced by ARP_WARP [46]. Model building, model completion and validation were then performed using the program Coot [47]. A final validation was done using MolProbity [48].
